# What is the optimal regimen for intravenous dexamethasone administration in primary total hip arthroplasty? A protocol of randomized controlled trial

**DOI:** 10.1097/MD.0000000000022070

**Published:** 2020-09-04

**Authors:** Dongliang Liang, Chao Xue, Weibo Liu, Yang Wang

**Affiliations:** Department of Orthopedics, The First Medical Center in PLA General Hospital, Beijing, 100853, China.

**Keywords:** dexamethasone, pain control, randomized controlled trial, study protocol, total hip arthroplasty

## Abstract

**Background:**

A number of recent studies have investigated the optimal dosage and timing of dexamethasone in total hip arthroplasty (THA) but have inconsistent findings. Therefore, we designed the randomized controlled research to look for the optimal intravenous dexamethasone dose for the treatment of early postoperative pain after the THA.

**Methods:**

The Declaration of Helsinki principles was followed and the Consolidated Standards of Reporting Trials guidelines for randomized controlled trials was adhered in this study. The First Medical Center in People's Liberation Army General Hospital approved the study (2020-089). After written informed consent was obtained, patients aged between 18 and 80 years with Physical Status I to III of American Society of Anesthesiologists, scheduled for primary unilateral THA, were included in this present work. Randomization is the use of a computer-formed list via a secretary, at a ratio of 1:1:1. The major end points were pain scores at 24 hours, 48 hours, and 72 hours after surgery, with visual analog scale (VAS) utilized at rest, and at 45 degrees passive hip flexion. The secondary outcomes involved the total consumption of morphine, opioid-related side effects, hip range of motion, inflammation markers, and the length of hospital stay.

**Results:**

We assumed that the patients who received 3 doses of dexamethasone intravenously possessed the best postoperative results compared to those who received 1 or 2 doses of the dexamethasone.

Trial registration: This study protocol was registered in Research Registry (researchregistry5864).

## 1 Introduction

Total hip arthroplasty (THA) is considered to be a economical, safe, and successful medical intervention, which can restore hip joint function and painless activity in the patients with trauma or serious joint diseases.^[1]^ Nevertheless, the inflammation induced by surgery often causes moderate to severe postoperative nausea and vomiting and pain, which brings great challenges to the early rehabilitation and recovery of patients. It can prolong the length of hospital stay, delay the recovery, and affect the satisfaction of patient.^[2–4]^

Dexamethasone is a powerful and long-acting glucocorticoid, which is widely utilized in the orthopedic perioperative period and has the effects of antiemesis and analgesia.^[5–7]^ Its mechanism of action is presumably mediated through a reduction in the production of inflammatory mediators, which may have benefits on pain and functional recovery.^[8–11]^ Nevertheless, owing to its latent side effects, the use of glucocorticoids in perioperative period is still controversial. Several researches have reported latent dexamethasone side effects, for instance, impaired sleep quality, increased infection risk, and elevated blood glucose early after surgery.^[6,12]^

However, owing to the clinical heterogeneity, the optimal dose and timing of dexamethasone in THA has not been determined, which may cause significant differences in the clinical outcomes. It is reported that a single dose of dexamethasone has the best effect in the first 24 hours.^[13,14]^ Nevertheless, the former researches reported that there was a pain peak within 72 hours after the THA.^[15,16]^ In Lei et al's study, the authors observed that the multiple low-dose dexamethasone can in-depth reduce postoperative pain, improve nausea after surgery, offer extra inflammatory control, increase activity, and decreased postoperative hospital stays.^[11]^ Therefore, we designed the randomized controlled research to look for the optimal intravenous dexamethasone dose for the treatment of early postoperative pain after the THA. We assumed that the patients who received 3 doses of dexamethasone intravenously possessed the best postoperative results compared to those who received 1 or 2 doses of the dexamethasone.

## 2 Materials and methods

### 2.1 Participants

The Declaration of Helsinki principles was followed and the Consolidated Standards of Reporting Trials guidelines for randomized controlled trials was adhered in this study. The trial was registered prior to patient enrollment via the Research Registry (researchregistry5864). The First Medical Center in People's Liberation Army General Hospital approved the study (2020-089). After written informed consent was obtained, patients aged between 18 and 80 years with Physical Status I to III of American Society of Anesthesiologists, scheduled for primary unilateral THA, were included in this present work.

The exclusion criteria involved revision surgery; prior ipsilateral hip surgery; exhibited sensitivity or allergy to dexamethasone, opioids, or any other drugs used in the study; ankylosing spondylitis; systemic lupus erythematosus; daily intake of strong opioids or dexamethasone; have a history of alcohol abuse or intravenous drug use.

### 2.2 Randomization and blinding

**Figure 1:**
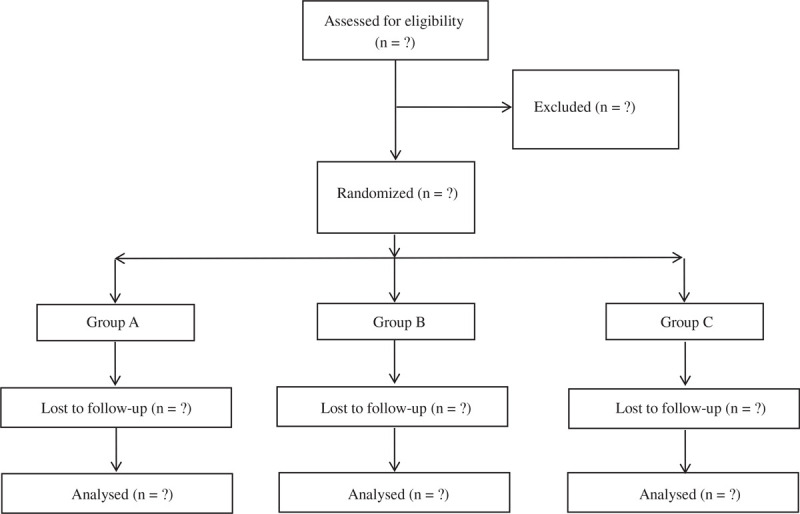
Flow diagram of the study.

Randomization is the use of a computer-formed list via a secretary (Research randomizer, www.randomizer.org), at a ratio of 1:1:1, each block has 50 numbers. Each participant received a serial research number from 1 to 150 and they also received treatment assigned in accordance with a randomized list. The list was kept and available to only 2 nurses preparing study medications. They do not interact with patients. All other outcome evaluators, participants, and clinicians were blind to this intervention. When all the selected patients completed this study, the randomization key was broken for the first time (Fig. 1).

### 2.3 Intervention measures

Patients in the group A were given 1 dose of dexamethasone (10 mg) intravenously before the anesthesia induction, and then 2 doses of the normal saline (2 ml) was added after 24 hours and 48 hours. Patients in the group B were given 1 dose of dexamethasone (10 mg) intravenously before the anesthesia induction, and 1 dose of dexamethasone (10 mg) after 24 hours, and afterward, these patients received another 1 dose of the normal saline after 48 hours. And patients in the group C were given 1 dose of dexamethasone (10 mg) intravenously before the anesthesia induction, and then 2 doses of the dexamethasone (10 mg) were added after 24 hours and 48 hours. The clinical staff who administered the medication were unaware of the composition of individual test preparation. All the researchers also conducted blind study on these 3 groups.

### 2.4 Intraoperative management

In the operating room, the patients inhaled oxygen through the mask to maintain blood oxygen saturation above 94%. General endotracheal anesthesia without adjuvant neuraxial analgesia was used for all patients. No long-acting opioid was administered intraoperatively. All the total hip arthroplasties were carried out via high-capacity surgeons with graduate training. We performed the operations without tourniquet application. All the patients received the THA with standard lateral method without cemented implants. No patients received local infiltration analgesia at any time during the intraoperative period.

### 2.5 Postoperative management

Under the assistance and supervision of physiotherapists, strength training and hip range of motion training were carried out. All the patients received the standard postoperative multimodal program of pain management. Postoperative analgesic regimen for the first 24 hours’ period included celecoxib 300 mg orally daily. In the 24 to 72 hours’ postoperative period, patients received celecoxib 200 mg orally every 12 hours. When patients reported the pain score >6 on the visual analogue scale (VAS) from 0 to 10 point, 10 mg of morphine was injected intramuscularly. Rescue antiemetics were administered with complaints of nausea and/or vomiting.

### 2.6 Outcome measures

The major end points were pain scores at 24 hours, 48 hours, and 72 hours after surgery, with VAS utilized at rest, and at 45 degrees passive hip flexion. The secondary outcomes involved the total consumption of morphine, opioid-related side effects, hip range of motion, inflammation markers, and the length of hospital stay, that is, the days from admission to discharge. The anti-inflammatory response was assessed with C-reactive protein and interleukin-6 values, these values were recorded at 24 hours, 48 hours, and 72 hours after the surgery. All outcomes were collected by members of the research team that were blinded to allocation of intervention.

### 2.7 Sample size calculation

The sample size of major endpoint was detected and it was calculated through utilizing the software of PASS 2011 (NCSS, LLC, Kaysville, UT). On the basis of the results of our former study, the VAS score of nausea after surgery in control group was 2.16. We expected a 0.72 difference in VAS score. When the power was 0.90, the significance level was 0.05, the sample size required calculation was 50 for each arm. In view of all the possible exclusion, we assigned 70 patients in each group.

### 2.8 Statistical analysis

All the statistical analyses are conducted via utilizing the SPSS v. 24 (IBM Corp., Armonk, NY). The descriptive statistics of clinical and demographic characteristics are expressed in terms of the mean standard deviation of serial scale variables. The difference between serial scale variables of normal distribution is detected with the Student's *t* test, whereas the non-normal variables are detected via utilizing the Wilcoxon rank sum test. The correlation between the categorical variables is detected via utilizing the Fisher's exact test or Pearson Chi-squared test. All the analyses are conducted based on the principle of intention-to-treat.

## 3 Discussion

Dexamethasone has potent anti-inflammatory effects, and has been used to reduce postsurgical pain and inflammation in a variety of conditions, including THA. It also has antiemetic properties and is commonly used for the prevention of postoperative nausea and vomiting. A number of recent studies have investigated the optimal dosage and timing of dexamethasone in THA but have inconsistent findings. Therefore, we designed the randomized controlled research to look for the optimal intravenous dexamethasone dose for the treatment of early postoperative pain after the THA. We assumed that the patients who received 3 doses of dexamethasone intravenously possessed the best postoperative results compared to those who received 1 or 2 doses of the dexamethasone.

## Author contributions

**Conceptualization:** Yang Wang.

**Data curation:** Dongliang Liang, Chao Xue.

**Formal analysis:** Dongliang Liang, Chao Xue.

**Funding acquisition:** Yang Wang.

**Investigation:** Dongliang Liang, Chao Xue, Weibo Liu.

**Methodology:** Dongliang Liang, Chao Xue, Weibo Liu.

**Resources:** Yang Wang.

**Software:** Yang Wang.

**Supervision:** Yang Wang.

**Validation:** Weibo Liu.

**Visualization:** Weibo Liu.

**Writing – original draft:** Dongliang Liang.

**Writing – review & editing:** Weibo Liu.
